# Body image concerns in individuals diagnosed with benign gynaecological conditions: scoping review and meta-synthesis

**DOI:** 10.1080/21642850.2021.1920949

**Published:** 2021-05-15

**Authors:** Katherine Sayer-Jones, Kerry A. Sherman

**Affiliations:** Department of Psychology, Centre for Emotional Health, Macquarie University, Sydney, Australia

**Keywords:** Scoping review, meta-synthesis, benign gynaecological conditions, body image, qualitative

## Abstract

**Background:**

Benign gynaecological conditions (BCGs) and body image-related concerns are commonly experienced by reproductive-aged female-identified individuals. Qualitative evidence from cancer populations identifies a link between diseases of the sexual organs and body image distress encompassing appearance, sensory and functional aspects. Most BCGs and the impacts on body image have been studied separately. However, commonalities exist between these conditions including chronicity, diagnostic delays, and menstrual-related social stigma. This systematic scoping review and meta-synthesis aimed to compare and contrast the experience of body image in the benign conditions of endometriosis, polycystic ovarian cysts, uterine fibroids, and vulvar intraepithelial neoplasia.

**Method:**

Electronic databases (MEDLINE, PsycINFO, Scopus, CINAHL, Embase, and Allied and Complementary Medicine) were searched in February 2020 and relevant articles were examined to identify papers that qualitatively explored the relationship between body image and BCGs. Meta-synthesis was used to analyse the 17 papers that met the inclusion criteria.

**Results:**

Six main themes evolved from this iterative analysis: loss of control; regained control; silence – menstrual concealment; cultural differences; feeling abnormal, and functional impairment. Body image concerns were widespread although impacts on individual’s lives were dependent on the unique symptom profile of each disease which interacted with socio-cultural factors, daily functioning, and feminine identity. Body image concerns were a common, but hidden, experience rarely screened in routine clinical settings despite causing significant distress.

**Conclusions:**

The chronicity and severity of individuals unique symptom profile often determined the intensity and type of body image concerns individuals described. Across conditions, body image concerns were often left untreated, were concealed, and were associated with reduced quality of life.

Benign gynaecological conditions (BGCs) including endometriosis, polycystic ovarian syndrome (PCOS), uterine fibroids, and vulvar intraepithelial neoplasia (VIN) are collectively common amongst reproductive aged female-identified individuals. Endometriosis is a condition where endometrial tissue develops outside the uterine cavity and is associated with pelvic pain symptoms (Fauconnier & Chapron, 2005). PCOS is a hormonal disorder associated with a profile of symptoms including menstrual irregularity, acne, obesity, and hirsutism (Rong et al., 2010). Uterine fibroids involve the growth of non-cancerous growths in the uterus with symptoms including heavy menstrual bleeding, protracted periods and pelvic pain (Stewart, 2001). VIN is a pre-cancerous skin condition of abnormal cells on the vulva, symptoms can include chronic irritation, skin appearance changes, and painful sexual contact (Hart, 2001). Prevalence rates vary across conditions, with 9–15% diagnosed with endometriosis (Giudice & Kao, 2004), 4–21% PCOS (Rong et al., 2010), and 4.5–17.5% uterine fibroids (Zimmermann, Bernuit, Gerlinger, Schaefers, & Geppert, 2012), and VIN 5 in every 100,000 (Bodelon, Madeleine, Voigt, & Weiss, 2009).

These BGCs differ in their complexity, impact on daily functioning, and symptom severity, ranging from absent to disabling (Jones, Kennedy, & Jenkinson, 2002). Each of these reproductive health concerns shares a similar socio-cultural context in that they belong within an ‘etiquette of menstruation’ requiring concealment (Johnston-Robledo & Chrisler, 2013; Seear, 2009), which contributes to diagnostic delays (Culley et al., 2013; Gibson-Helm, Teede, Dunaif, & Dokras, 2016). Anxiety, depression, and diminished quality of life are commonly reported by individuals with BGCs (Dokras, Clifton, Futterweit, & Wild, 2012; Marinho et al., 2018; Moghadam, Fereidooni, Saffari, & Montazeri, 2018; Williams, Jones, Mauskopf, Spalding, & Duchane, 2006), with treatment delays further exacerbating these mental health concerns (Grace & MacBride-Stewart, 2007; Kennedy, 1991).

Another critical psychosocial issue for individuals living with BGCs is that of body image, reflecting the interplay between thoughts, feelings, and perceptions about the whole body, and its functioning (Alleva & Tylka, 2021; Fingeret et al., 2012). Acute or chronic illnesses, such as BGCs, can result in the subjective re-evaluation of one’s bodily function and appearance (Cash, 2012; Fingeret, 2010). Hence, BGCs may impact on body image aspects related to appearance (e.g. abdominal bloating, weight gain, acne), sensory perception (e.g. pain, numbness, and physical heaviness) and function (e.g. dyspareunia, excessive bleeding, and infertility) (Fingeret et al., 2012). Yet body image experiences do not necessarily reflect objective changes to the body (Pruzinsky, 2004) or align with clinical severity of the condition (Rumsey & Harcourt, 2004). Research on BGCs is of importance as while the literature indicates a link between body image concerns in female-identified bodies with oncological conditions such as breast cancer (Davis et al., 2020), benign conditions have not received the same research attention.

Since BGCs disproportionally affect female-identified individuals of reproductive age, whose identity is typically contingent on physical appearance and aspects of intimate relationships (e.g. frequency and quality of sexual intimacy and family planning; Moradi, Parker, Sneddon, Lopez, & Ellwood, 2014), impact on body image is particularly salient. Research in the field of eating disorders and body image highlights the reciprocal relationship concerning the body and culture (Gattario, Frisén, Teall, & Piran, 2020). Especially how living in a feminised body can constraint interactions with the health system (Piran, 2019). Body image concerns can result in psychological distress akin to clinical social anxiety, with symptoms of fear and avoidance of social situations and reduced functioning (American Psychiatric Association, 2013; Kent, 2000; Newell & Marks, 2000). BGCs can impact body image in diverse ways. For example, in PCOS outwardly visible aspects of the condition including hirsutism and weight control disrupt feminine identity and body image (Azziz, 2006; Bazarganipour et al., 2015; Hollinrake, Abreu, Maifeld, Van Voorhis, & Dokras, 2007; Kitzinger & Willmott, 2002; Pfister & Rømer, 2017). For other conditions where bodily changes may be less outwardly visible (e.g. endometriosis), body image concerns can adversely impact interpersonal and sexual relationships (Ballweg, 2011; Nnoaham et al., 2011; Tripoli et al., 2011). Moreover, common to all the BGCs, is the chronicity and uncertainty regarding the condition’s progression and future impacts on bodily appearance, sensory symptoms and functioning (e.g. infertility) (Crete & Adamshick, 2011; Lemaire, 2004).

Given the nature of body image concerns related to women’s reproductive health, qualitative research methodologies offer real-world insight into how individuals navigate these complex disorders (Cox, Henderson, Andersen, Cagliarini, & Ski, 2003; Denny & Khan, 2006). Yet, despite the commonalities across the different BGCs, most qualitative research has focused on a single disease population (e.g. endometriosis – Young, Fisher, & Kirkman, 2015; PCOS – Moghadam et al., 2018) and there is an absence of integrated reviews of this literature. The aim of this scoping review was to address this gap by integrating qualitative studies through examining body image concerns of individuals with BGCs, enabling a broader understanding of how individuals contextualise their BGCs within their social world (Green & Thorogood, 2018). The range of common BGCs in this synthesis included endometriosis, PCOS, uterine fibroids, and VIN, all of which are characterised by chronicity, delays in diagnosis, and being a ‘menstrual’ condition (Jones et al., 2002; Seear, 2009). A ‘meta-ethnographic’ method of analysis/synthesis was adopted for this integration of qualitative research findings (Atkins et al., 2008; Noblit & Hare, 1988); an approach that is particularly valuable in health care research when the purpose is to ascertain aspects of quality of life in the context of illness (Campbell et al., 2003; Shaw, Sherman, & Fitness, 2015). This scoping review aimed to identify key qualitative themes regarding body image impacts, whilst ascertaining the differences and similarities in lived experience across BGCs, and to identify gaps in the literature.

## Methodology and method

### Search strategy

In February 2020, a systematic literature search of MEDLINE, PsycINFO, Scopus, CINAHL, Embase, and Allied and Complementary Medicine was conducted to identify all qualitative studies on the topic of body image amongst female-identified individuals with BGCs using the terms: ‘endometriosis’, ‘gynaecological’, ‘menstrual disorders’, ‘pelvic pain’, ‘dyspareunia’, ‘polycystic ovarian syndrome’, ‘endocrine sexual disorders’, ‘hysterectomy’, ‘pelvic inflammatory disease’, ‘menstrual’, ‘menstrual cycle’, ‘inflammation’, ‘vaginismus’, ‘uterus’, ‘fibroids’, ‘reproductive health’, ‘benign’, ‘menstrual cycle’, ‘abdominal bloating’, ‘infertility’, ‘urogenital disorders’, combined with an ‘OR’ search of ‘body image’, ‘body awareness’, ‘body dissatisfaction’, ‘body satisfaction, ‘body appreciation’, ‘body weight’, ‘body shape’, ‘body esteem’, ‘body concern’, ‘body appearance’, ‘weight concern’, ‘body disturbance’, combined with an ‘OR’ search of ‘interview’, ‘narrative’, and ‘qualitative’.

### Study selection and quality appraisal

**Figure 1.**
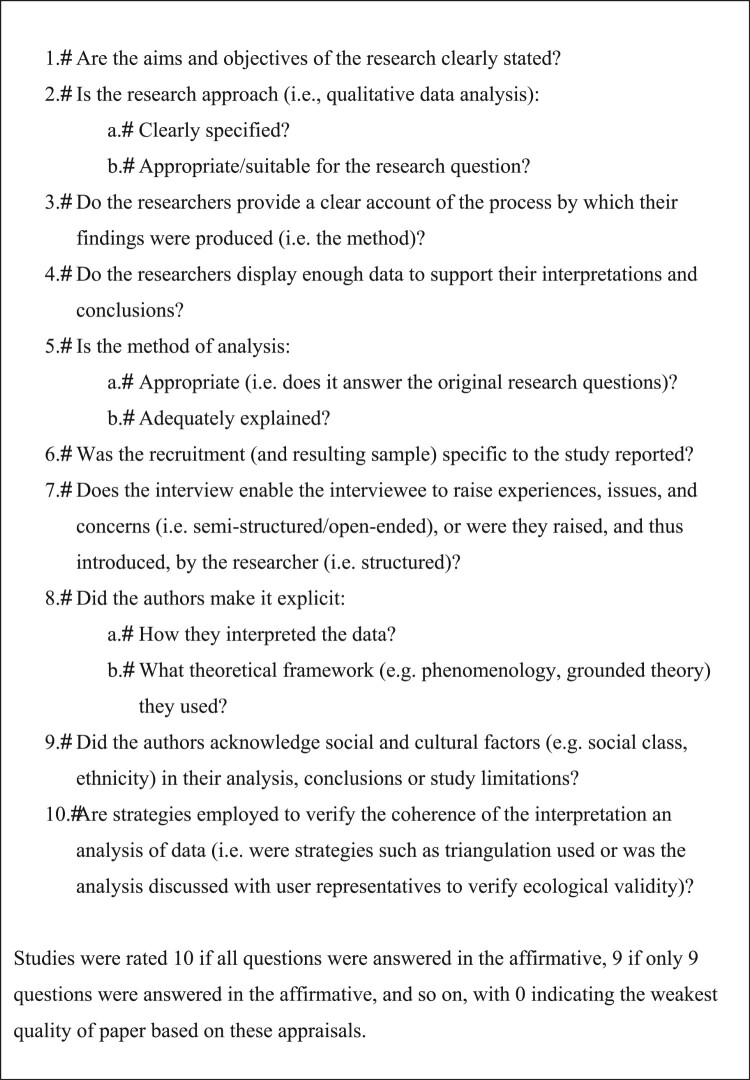
Quality appraisal criteria.

**Figure 2.**
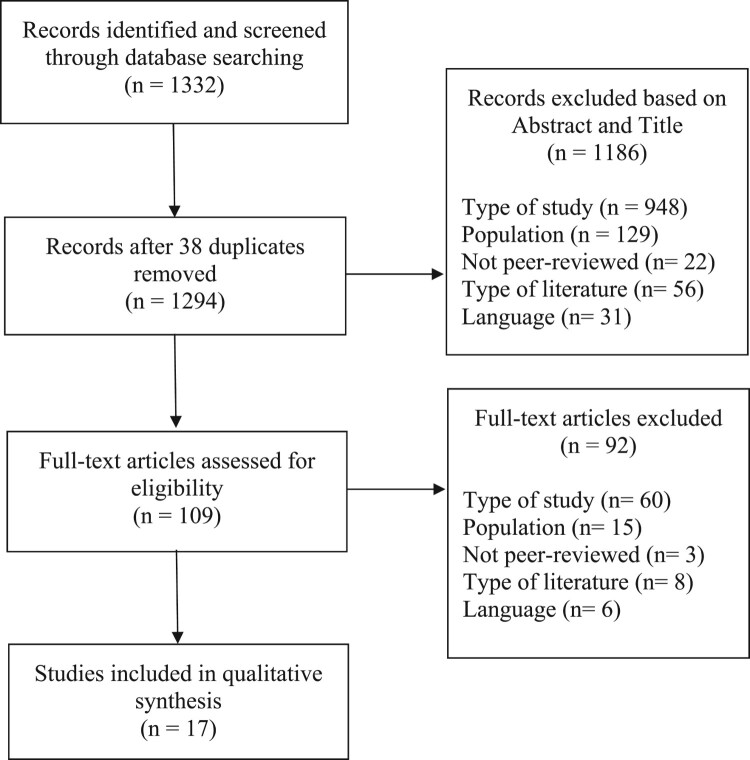
PRISMA diagram of search strategy.

Studies meeting the following criteria were included: (1) used qualitative methodology; (2) published in an English-language peer-reviewed journal; (3) involved female-identified participants over the age of 17 years who were diagnosed by a health professional with a BGC; and, (4) included explicit or conceptual reference to body image in the study results. Discussions, editorials, reviews, commentaries, and dissertations were excluded, as were studies focused primarily on malignant conditions, urinary tract conditions (e.g. incontinence), pregnancy (e.g. ectopic pregnancy), or disorders of sex development. Grey literature, defined as research that had not undergone peer-review at the date of the systematic search was also excluded (Pappas & Williams, 2011). Studies were vetted and excluded when the title and abstract indicated they were obviously unrelated to the research question or they fulfilled the exclusion criteria. Full-text versions of studies were accessed when the title and abstract had relevance to the topic. Papers that fulfilled the inclusion criteria were subjected to the meta-synthesis analysis as well as an evaluation of the study’s quality based on previously published criteria (see Figure 1; Adams et al., 2011; Dixon-Woods et al., 2006; Shaw et al., 2015), since deficient methodologies or designs risked the secondary interpretation of these data (Lang, France, Williams, Humphris, & Wells, 2013). Record of this process was documented in Figure 2 accordance with the PRISMA guidelines (Panic, Leoncini, De Belvis, Ricciardi, & Boccia, 2013). Ethics approval was obtained from Macquarie University Ethics Committee.

### Meta-synthesis process

Noblit and Hare’s (1988) interpretive approach was applied to compare, reanalyse and integrate the findings of individual qualitative studies. This process involved three stages (Lang et al., 2013): (1) extraction of core characteristics of each study; (2) creation of interpretive metaphors, discussion and refinement; and (3) comparison of metaphors and themes of BCG phenomena within each study. This analysis was an iterative process where the individual findings of included studies were compared and contrasted.

## Results

The outcomes of the search strategy are provided in the Prisma Diagram (Figure 2). From an initial search yield of 1332 studies, after applying inclusion/exclusion criteria, 17 published qualitative studies were included in this review. The overall quality of papers was represented in a ‘summed score’ with higher numbers indicating greater research quality based on guidelines developed in prior research (Adams et al., 2011; Dixon-Woods et al., 2006). The quality ratings for all 17 included studies are provided in Table 1, with the majority of studies being a high standard based on these criteria (most scored above 8.5 out of a possible 10). Notably, quality was reduced in several papers due to the absence of theory in the qualitative analysis and lack of socio-cultural consideration.

**Table 1.** Quality appraisal criteria.

Author	Aims	Approach	Account	Data	Analysis	Recruitment	Structure	Theory	Socio-cultural	Validity	Score
Facchin et al. (2018)	1	1	1	1	1	1	1	1	1	1	10
Gonçalves et al. (2016)	1	1	1	1	1	0	1	0.5	1	1	8.5
Ghant et al. (2015)	1	1	1	1	1	0	1	1	1	1	9
Grundström et al. (2018)	1	0.5	1	1	1	1	1	1	0	1	8.5
Hållstam et al. (2018)	1	1	1	1	1	0	1	1	1	1	9
Hudson et al. (2016)	1	1	1	1	1	1	1	1	1	1	10
Jones et al. (2011)	1	1	1	1	1	1	1	0	0	0	7
Kitzinger and Willmott (2002)	0	1	1	1	0.5	1	1	1	1	0	7.5
Likes et al. (2008)	1	1	1	1	1	1	1	0	0	1	8
Lim et al. (2019)	1	1	1	1	1	1	1	1	1	1	10
Markovic et al. (2008)	1	1	1	1	1	0	1	1	1	1	9
Sharma and Mishra (2018)	1	1	1	1	1	1	1	1	1	1	10
Thorpe et al. (2019)	1	1	1	1	1	1	0	1	0	1	8
Tomlinson et al. (2017)	1	1	1	1	0.5	1	1	0.5	0	1	8
Washington (2008)	1	1	1	1	1	1	1	0	1	1	9.5
Wu et al. (2014)	1	1	1	1	1	1	1	1	1	1	10

Notes: Ratings are for quality appraisal criteria detailed in Figure 1, scores of 1 = meets criterion, 0.5 = partially meets criterion, 0 = does not meet this criterion. Overall quality of paper represented in ‘score’, with higher numbers indicating greater research quality based on guidelines from previous research (Adams et al., 2011; Dixon-Woods et al., 2006).

The details and characteristics of each included paper are outlined in Table 2. Of the 17 included studies, the sample size ranged from 6 to 83, and age ranged from 17 to 72 years. The types of BGCs studied included endometriosis (*n *= 5), PCOS (*n *= 8), uterine fibroids (*n *= 3) and VIN (*n *= 1). The meta-synthesis yielded six main areas of body image concern: loss of control; regained control; silence – menstrual concealment; cultural differences; feeling abnormal, and functional impairment. The body image concerns and challenges raised by women with different BGCs were largely similar, hence the identified themes are integrated together within the Results section, with specific examples and symptom profiles represented. Any identified themes and extracted direct quotes indicate the type of diagnosis from which the qualitative data originated. Notably, the regaining control theme was more representative of those who had undergone surgical intervention that abated their symptoms. Additionally, the cultural differences theme represented a facet of studies on fibroids and PCOS.

**Table 2.** Study characteristics.

Author	*N*	Population			Approach			Themes^a^
		Gender, (age range)	Country/ethnicity	Diagnosis	Data	Analysis	Theory	
Facchin et al. (2018)	74	Female (24–50)	Italy	Endo	F2F Interview	Grounded	Grounded	Pathway to diagnosis; Quality of doctor-patient relationships; Current presence of symptoms; Support; Female identity; Meaning of life with endometriosis
Gonçalves et al. (2016)	15	Female (24–49)	Brazil	Endo	F2F Interview	Framework Analysis Method	None reported	Expectations of yoga; Physical and emotional state; Control and pain; Secondary benefits; Self-knowledge; Psychosocial support
Ghant et al. (2015)	60	Female (29–55)	White Black Asian Hispanic	Fibroids	F2F Interview	Grounded	Grounded	Negative body image and sexuality; Psychological distress; Lack of support; Strengths and limitations; Helplessness
Grundström et al. (2018)	9	Female (25–55)	Unknown	Endo	F2F Interview	Stevick–Colaizzi–Keen Method	Phenomenological	Double edged sword of experience; Ignorance; Acknowledgement
Hadjiconstantinou et al. (2017)	12	Female (17–51)	White Black Asian	PCOS	F2F Interview	Policy Research	None reported	Symptoms and diagnosis; Psychological distress; Practical implications; Coping support needs
Hållstam et al. (2018)	13	Female (24–48)	Sweden Europe	Endo	F2F Interview	Grounded	Grounded	'I’m different’; Dependence; Ruined Life; Struggle for coherence
Hudson et al. (2016)	44	Female (25–50) and male (26–57)	White South Asian Other	Endo	F2F Interview	Thematic	Interpretative relational paradigm	Sex and intimacy; Children; Working lives and social lives
Jones et al. (2011)	15	Female (17–21)	White Mixed	PCOS	F2F Interview	Thematic	None reported	Knowledge of PCOS; Hirsutism; Weight; Acne; Menstrual problems; Infertility; Emotions; Personal relationships; Sexual relationships; Professional help; Diet and exercise; Self-perception; Positive aspects
Kitzinger and Willmott (2002)	30	Female (21–42)	White Non-White	PCOS	F2F Interview	Discursive analysis	None reported	Bearded ladies and hairy monsters; Irregular women; Infertility
Likes et al. (2008)	6	Female (22–72)	White	VIN	Focus groups	None listed	None reported	Spirituality and time in life; Significant others; Health care providers; Others with VIN
Lim et al. (2019)	10	Female (18–45)	White Asian, Oceanic Other	PCOS	F2F interview	Thematic	Grounded	Weight loss journey; PCOS on weight; Past weight loss experience; Facilitators to management; Barriers
Markovic et al. (2008)	30	Female (30–60+)	Not reported	Fibroids	F2F interview	Thematic	Contextualism	Hysterectomy as loss; Hysterectomy as gain; Health and social life
Sharma and Mishra (2018)	35	Female (not reported)	Indian	PCOS	F2F interview	Interpretative phenomenological analysis	Phenomenology	PCOS as a tabooed disease; Infertility; Lack of awareness; Social construction
Thorpe et al. (2019)	83	Female non - binary (19–43)	Not reported	PCOS	Drawings, text-based format	Thematic	Critical visual methodology	Awareness of visible and invisible symptoms; Misshapen self and body; Limitations and resignation
Tomlinson et al. (2017)	32	Female (18–42)	Not reported	PCOS	Focus Groups	Grounded	None reported	Diagnostic delays; Lack of empathy; Accessing specialist referral; Lack of information; Inconsistent advice; Insufficient help in/fertility; Longer term risks; Discrepancies in beliefs
Washington (2008)	11	Female (25–39)	Caucasian African-American Native American	PCOS	2 x F2F interviews	None reported	None reported	Pain; Impact on self-concept; Excessive hair and hair loss; Uncontrolled emotions as a Result of PCOS; Impact on workplace; Conception; Employment
Wu et al. (2014)	17	Female (37–50)	Taiwan	Fibroids	F2F interviews	Content	Bounded rationality	Release from stress; Inescapable fate; Positive support; Peace of mind and trust

^a^Theme descriptions have been shortened for brevity.

### Theme: loss of control

All 17 studies reviewed indicated that the presence of a BGC was associated with loss of control. Uncontrollable unwanted physical changes were proportional to the individual’s description of losing control of multiple aspects of their body image including their appearance (e.g. weight gain, facial hair; Thorpe, Arbeau, & Budlong, 2019), sensory pain-related changes (Gonçalves, Makuch, Setubal, Barros, & Bahamondes, 2016), and threats to their functional integrity (e.g. in/fertility heavy bleeding, benign tumour growth; Ghant et al., 2015; Markovic, Manderson, & Warren, 2008; Tomlinson et al., 2017):

It just makes me kind of feel like there is nothing I can do about it you know, something is taking over my body that I have no control over. (Fibroids; Ghant et al., 2015)

This loss of control was associated with a sense of helplessness for those who underwent invasive surgical procedures only to have their symptoms reoccur, such as laparoscopy for endometriosis (Grundström, Alehagen, Kjølhede, & Berterö, 2018), or surgeries on the labia with VIN (Likes, Russell, & Tillmanns, 2008):

They cut it out and it’s all gone and you’re good for that day. The next day you get up, it can be right back. (VIN; Likes et al., 2008)

When I did the surgery and then felt all that pain again it was very frustrating. It felt as if I was swimming hard in the ocean just to end up dying at the beach. (Endometriosis; Gonçalves et al., 2016)

A major concern for individuals with PCOS was the inability to lose weight (Jones, Hall, Lashen, Balen, & Ledger, 2011; Lim et al., 2019; Tomlinson et al., 2017), creating a mismatch with the cultural thin ideal (Kitzinger & Willmott, 2002). Here, individuals used pejorative language to describe their body:

I was always somebody who felt like I had control over my body I suppose and so suddenly there was something that I couldn’t control any more. And I suppose linked to that, yes, just the weight thing is just – I look at, even now, I look at myself in the mirror and I think “Ugh, not good”. (PCOS; Hadjiconstantinou et al., 2017)

Body image distress and helplessness associated with endometriosis and PCOS increased when there were inadequate treatment options offered by health professionals, placing the burden of responsibility to change body image concerns on the woman herself (Grundström et al., 2018). Individuals described these interactions as a degrading form of commentary as opposed to intervention:

I was given advice like ‘start exercising or something’. It made me feel as if I was being ridiculed. (Endometriosis; Grundström et al., 2018)

They … said I had polycystic ovaries and I might struggle having children and that I was really high risk of having diabetes with my weight … basically, all they said to me was lose weight. (PCOS; Tomlinson et al., 2017)

Being dismissed in this way was incongruous with many women’s motivation for change. Individuals described being proactive in their efforts to gain a sense of mastery in managing their conditions by having, ‘Tried everything’ including diet, exercise and different types of alternative therapies to no avail (Lim et al., 2019). Many women reported limited knowledge of how to manage future health consequences of their BGC. Lack of control was also evident due to financial barriers or material circumstances of their lives (Lim et al., 2019). Women’s ability to regain a sense of confidence and control was often contingent on health professional-led management, including psychological intervention (e.g. individuals with endometriosis described learning to manage expectations and carefully plan social events to accommodate fatigue and pain; Hållstam, Stålnacke, Svensén, & Löfgren, 2018).

### Theme: regained control

Individuals in six of the studies investigating fibroids, endometriosis and PCOS found ways to regain a sense of control in their lives based on the availability of treatments (e.g. removal of reproductive organs) for their diagnosis and individual proclivity to psychologically cope (Facchin, Saita, Barbara, Dridi, & Vercellini, 2018; Ghant et al., 2015; Markovic et al., 2008; Wu, Lee, Yeh, & Che, 2014). Once these individuals grieved this symbolic loss of femininity and functional changes to their bodies, the surgical decision welcomed a relief of BGC symptoms:

… it was a liberation … because it meant no more PMT (premenstrual tension), no more MT (menstrual tension), no more after-PMT (postmenstrual tension), no more pain, a huge financial saving. A hell of a lot less distress. I mean it was just a liberation … absolutely. (Fibroids; Markovic et al., 2008)

A sense of control was also gained by learning how to psychologically accommodate their BGC as opposed to denying reality, with less body image distress experienced for those who intentionally created routine and mastery in their daily activities, which positively impacted their relationship with their endometriosis and bodies overall:

I am satisfied with my body, I control what I eat, I exercise, I try to take care of myself as much as I can […] but overall I have a positive relationship with my body. (Endometriosis; Facchin et al., 2018)

Women with endometriosis in one study described learning to meticulously plan social events to allow for fatigue or pain as means of gaining control (Hållstam et al., 2018). Although the BGC symptoms remained unpredictable, these coping strategies enabled individuals to develop a sense of personal mastery that accentuated their sense of control as opposed to feeling psychologically overwhelmed:

When I saw that I could manage, when I decided there’d be no excuses, then I got my life back … I did not want to end up in that downward spiral again. (Endometriosis; Hållstam et al., 2018)

Improved body image was often associated with an improved understanding of their physical condition through communications with a helpful health care professional that aided faciliatory coping strategies (e.g. techniques to better manage endometriosis pain; Grundström et al., 2018). Regained control was also identified by women who had their BGC experiences and distress normalised within their community through the sharing of personal experience, opinion, and/or a non-judgemental stance (Gonçalves et al., 2016; Wu et al., 2014):

My mother-in-law said that I should not think about it so much. There are a great many women who have had their uterus removed, so I should not worry too much about it. (Uterine fibroids; Wu et al., 2014)

### Theme: silence – reproductive concealment

Across all BCGs, ten studies indicated individuals engaged in concealment practises of their condition, with symptoms degrading the experience of womanhood (e.g. in PCOS subverting physical representations of idealised feminine body image; Markovic et al., 2008), and compromising the ‘legitimate’ female status to themselves and/or in the eyes of others (Grundström et al., 2018; Hudson et al., 2016; Markovic et al., 2008; Wu et al., 2014). The invisible BGC concern of infertility disrupted the reproductive narrative (Hållstam et al., 2018; Kitzinger & Willmott, 2002; Sharma & Mishra, 2018; Thorpe et al., 2019; Tomlinson et al., 2017; Washington, 2008):

I avoid saying it [about irregular cycle] to people because I think they would see me as not a proper woman. (PCOS; Kitzinger & Willmott, 2002)

Their BGC was said to stigmatise, with affected individuals perceived as undesirable (‘unruly’, ‘freakish’; Kitzinger & Willmott, 2002), particularly if their efforts to conceal symptoms failed (Hadjiconstantinou et al., 2017):

You know, people, when they’re looking at your face you think ‘Oh God, are they looking at the hair? Are they looking at my moustache?’ And you’re covering your face and, trying to hide it and you’re actually drawing more attention to it (PCOS; Thorpe et al., 2019)

Stigma maintained the pressure to conceal symptoms, particularly aesthetically (Hadjiconstantinou et al., 2017; Kitzinger & Willmott, 2002) with some studies (Ghant et al., 2015; Kitzinger & Willmott, 2002) reporting that different BGC symptoms elicited a range of responses ranging from compassionate to degrading:

I am much happier about, umm, disclosing the sort of fertility implications … People respond and, you know, and then it’s like, there’s sympathy. You know, obviously that a woman would be distressed about that. Whereas I’m just not sure what the reaction would be if I talked about growing a beard or a moustache. (PCOS; Kitzinger & Willmott, 2002)

Pressure to conceal their condition due to the association with menstrual-related concerns resulted in hypervigilance for some women who described anticipatory stress:

You're constantly worried about you know you're taking public transportation to work so you don't want to stand up and get that rush and now you're embarrassed because you know you've had an accident that you couldn't control, it just happens and there's no way to plan for it. (Uterine fibroids; Ghant et al., 2015)

Propensity for concealment also reflected women’s distress that friends and family would normalise their experience and minimise the severity of their suffering (Ghant et al., 2015; Likes et al., 2008):

They don’t see what I’m going through, the nights when I would start flooding. (Uterine fibroids; Ghant et al., 2015)

For many, the normalisation of their benign conditions beneath the umbrella terms of menstruation was a common experience, and therefore some avoided the topic without question to meet this expectation and self-protect. For some, avoidance and concealment of menstrual-related symptoms were self-protective (Hållstam et al., 2018), being reserved for discussion with professionals (Wu et al., 2014), or intimate partners (Hudson et al., 2016).

### Theme: cultural differences

Five studies highlighted the challenges experienced by women from ethnically diverse groups with PCOS and fibroids, with stigma related to their gendered roles as a wife and mother (Hadjiconstantinou et al., 2017; Markovic et al., 2008; Sharma & Mishra, 2018). These individuals described pressure to bear children and suppression of discussion about menstrual-related topics. Cultural expectations heightened concerns that their BGC would limit their social acceptability, create problems for themselves and their family, and produced further pressure to conceal symptoms (Hadjiconstantinou et al., 2017; Hudson et al., 2016; Markovic et al., 2008; Sharma & Mishra, 2018; Wu et al., 2014). Women from India indicated that PCOS was highly concealed because menstrual issues were a taboo subject and fertility is considered integral to a women’s function and familial social standing in traditional Indian society (Sharma & Mishra, 2018):

My mother-in-law told me that I should not tell anyone that I had irregularity of periods since its onset. People would say that I was defected from the beginning. (PCOS; Sharma & Mishra, 2018)

BGC’s acted as a barrier to this pre-ordained societal role from occurring and opened individuals up to being ostracised:

Amongst Asian couples if you can’t have a child, it’s almost like you're a waste of space, they don’t want to have a daughter-in-law or a wife who can’t have a baby. (PCOS; Hudson et al., 2016)

In a study of Taiwanese women with fibroids, hysterectomy was acceptable as those who already had children could prioritise treating their acute symptoms of heavy bleeding and risk of malignancy (Wu et al., 2014). Ethnically diverse individuals in the United Kingdom indicated that some women’s culture as well as their environmental norms shaped their condition’s symptoms as stigmatised, such as pregnancy, compared to facial hair (Hadjiconstantinou et al., 2017). As one participant with African heritage indicated:

… it’s not normal for a woman not to, to have (children), especially in African cultures it’s like taboo … But with the facial hair, in Africa it’s okay because a lot of women have it. It’s just here in England … you have to shave your legs and you have to shave this and that, when back home it’s not like that. (PCOS; Hadjiconstantinou et al., 2017)

### Theme: feeling abnormal

Of 12 papers reviewed, individuals across all BGCs examined shared concern that their symptoms made them feel abnormal, due to the perceived transgression of womanhood and negative stereotypes associated with symptoms, particularly when comparing with non-BGC affected female friends and family (Ghant et al., 2015; Thorpe et al., 2019):

I used to get quite down with that (hirsutism), because it was something, I’ve got sisters as well and obviously even comparing myself to my sisters they didn’t have anything like that and it really did upset me. (PCOS; Hadjiconstantinou et al., 2017)

I still feel like there's less of me because of this … all my friends are pregnant. (Uterine fibroids; Ghant et al., 2015)

Subsequently, many individuals desired normality (Thorpe et al., 2019), seeing their body ‘as other’, and perceiving that the presence of BGC symptoms meant that they would be evaluated in pejorative ways by others:

The side effects like the weight gain … really easy way to discriminate against somebody because you might assume that they’re lazy or that they’re unmotivated … (PCOS; Washington, 2008)

This negative evaluation extended to women’s intimate lives, resulting in self-consciousness, reduced sexual enjoyment and the feeling of not having ‘a normal sex life’ (Hudson et al., 2016):

It affected my sexual life because I didn't care for my partner to see me naked because I felt my shape would look so bad. It was embarrassing. I was uncomfortable and so it affected my self-esteem a lot. (Uterine fibroids; Ghant et al., 2015)

Symptoms such as PCOS-related hirsutism led to fear of ridicule via norms that allowed the culturally sanctioned degradation of women with stereotypical masculine features (Hadjiconstantinou et al., 2017; Kitzinger & Willmott, 2002; Tomlinson et al., 2017):

A woman with a beard is, you know, terrible. […] I think the hair is the worst thing because it’s visible … and quite often there are jokes on the telly about women with moustaches or beards and everything, and I go cold when that happens. (PCOS; Kitzinger & Willmott, 2002).

Individuals expressed concern that negative impacts of their BGC extended to their whole self-image, impairing the ability to have a positive relationship with one’s body and oneself:

PCOS is always described as being seated in the reproductive system but it extends its influence across my whole body. It feels like tendrils slowly choking every part of me. Everywhere it takes hold I feel a bit more broken and sad. My body doesn’t feel like it’s mine anymore and fighting it sometimes seems hopeless. (PCOS; Thorpe et al., 2019)

There’s only one thing I like of my body: my hair. I would throw away everything else. (Endometriosis; Facchin et al., 2018)

Corresponding to the diagnosis of depression, individuals in several studies reported feeling depressed, reporting low mood, diminished pleasure in activities, low self-worth, and fatigue (Hållstam et al., 2018; Likes et al., 2008; Sharma & Mishra, 2018; Washington, 2008).

### Theme: functional impact

Individuals across nine studies and all BGCs (Facchin et al., 2018; Ghant et al., 2015; Hållstam et al., 2018; Kitzinger & Willmott, 2002; Likes et al., 2008; Markovic et al., 2008; Sharma & Mishra, 2018; Thorpe et al., 2019; Wu et al., 2014) voiced concern that their condition had a functional impact on their daily lives practically, psychologically, and in their relationships:

But I feel … limited is the word, I think. You feel limited and, not free somehow … You sort of want to do things and be active and that, that part of me is no longer there … So it’s taken away much of the spark of life, my joy of living and that. (Endometriosis; Hållstam et al., 2018)

Diminished reproductive status (Hudson et al., 2016; Markovic et al., 2008; Sharma & Mishra, 2018; Thorpe et al., 2019) adversely impacted psychological functioning and relationship formation and quality of long-term partnership (Ghant et al., 2015; Kitzinger & Willmott, 2002; Likes et al., 2008; Markovic et al., 2008; Sharma & Mishra, 2018; Wu et al., 2014). Several studies indicated that infertility meant that affected individuals did not meet the cultural expectations of women resulting in social deprivation (Sharma & Mishra, 2018):

My mother-in-law treats me like a domestic maid. She says that I am good for nothing as I couldn’t provide them with a grandchild, so I can at least be useful in the domestic chores of the family. (PCOS; Sharma & Mishra, 2018)

Other elements of intimate lives were impacted such as pain from endometriosis reducing the enjoyment of sex (Hållstam et al., 2018; Markovic et al., 2008).

Management of symptoms could be embarrassing and inconvenient when they interfered with desired activities:

… you know, you’re at work, you go to the bathroom, you change all your pads and everything, and just 5 min later you have an accident in your chair. How do you get blood out of your chair at work? (PCOS, Washington, 2008)

For women with endometriosis, physical disability was more prevalent due to chronic pelvic pain (Hållstam et al., 2018):

I gave up my job because I couldn’t cope. And although he understood that, I kind of felt like I’d failed … luckily, he’s in a secure job, you know, and all that goes with that, but I was like putting extra sort of like financial burdens on the family and things like that. (Endometriosis; Hudson et al., 2016)

In contrast, a surgery that diminished symptoms, led to re-establishment of functional capacity and freedom to re-establish family and work roles (Markovic et al., 2008). BGC-related dysfunction and sick roles damaged the sense of self and the affected individuals’ ability to date and have a partner (Hållstam et al., 2018; Likes et al., 2008):

He said: I see no future for us and, er … you’re sick and things don’t look very bright, he said. Er: I want to be able to, I want to be together with a girl who I can go running with and skiing with and do things together with. And you can’t do these things. (Hållstam et al., 2018)

## Discussion

This meta-synthesis reviewed 17 qualitative studies, covering the common diagnosable gynaecological conditions of PCOS, endometriosis, uterine fibroids, and VIN. For most, being affected with a BGC is distressing, and a range of body image concerns are evident. Across the reviewed studies and BGC conditions, these concerns reflected the theoretical domains of body image in the context of illness identified by Fingeret (2010), whereby a BGC diagnosis activates re-evaluation of one’s bodily functioning and appearance, leading to body image deficits relating to appearance (e.g. excessive hair growth in PCOS), sensory changes (e.g. pain in endometriosis), and functional integrity (e.g. infertility). This analysis identified body image-related themes reflecting: loss of control (of internal and external body symptoms); regained control (based on treatment outcomes and psychological resilience); silence and concealment; cultural differences (related to symptom taboos); feeling abnormal (compared to individuals without BGCs); and, BGC-related impairments to full participation in daily life.

The loss of control theme related to aspects of physical appearance and function in the face of wide-ranging and variable symptoms, and perceptions of whether these were controllable or manageable. These findings are consistent with a review of chronic conditions more broadly (Hagger & Orbell, 2003), which identified perceptions of poor control over symptoms as being related to decreased social functioning and psychological adaptability (Leventhal, Meyer, & Nerenz, 1980). This theme also reflected dissatisfaction with healthcare provision characterised by diagnostic delays, lack of information, and vague lifestyle advice to manage their condition. Despite motivation to address body image concerns, many studies identified that affected individuals experience a sense of isolation and believe that healthcare professionals did not take their symptoms seriously and were unable to provide adequate referral pathways for addressing their body image concerns, consistent with quantitative research identifying extensive unmet needs regarding healthcare services and treatment options for individuals with BGCs (Gibson-Helm, Lucas, Boyle, & Teede, 2014).

In contrast to accounts of losing control, the theme of regained control represented how individuals had overcome BGC challenges via surgery or coping strategies. Treatments that alleviated acute suffering were seen to be helping restore a healthy body image by removing the functional impacts of disease (Williams & Clark, 2000). Additionally, regained control was associated with quality healthcare experiences where good communication about BGC symptoms and experiences was critical to ensuring a sense of being heard and supported. Clinical contexts providing supportive communications enabled a sense of self-efficacy and empowerment over the varied bodily impacts of the BGCs, particularly endometriosis. For affected individuals, personal acceptance and being honest with oneself were regarded as key in alleviating body image-related symptoms across these BGCs. Additionally, coping improved when individuals sought and received support (e.g. family, or a trusted doctor), reinforcing research indicating that discussion of disease, in general, can reduce loneliness and anxiety (Henry, Ekeroma, & Filoche, 2020). These supportive communications helped normalise the body-related concerns of affected individuals, resulting in the alleviation of body-related shame. Notably, individuals with PCOS were less represented in this theme.

Decisions about whether, or how, to disclose or conceal intimate gynaecological information to others were imbued in the Silence – reproductive concealment theme. The BGC’s stigmatised symptoms demanded diverse concealment practices and appraisal of the benefits versus costs of symptom disclosure (Chaudoir & Fisher, 2010). Specifically, infertility disrupted the reproductive narrative and risked negating individuals’ feminine identity, thus some limited these conversations to partners and health professionals. This supports findings that unless disclosure is considered necessary or the reciprocal reaction is supportive, those with stigmatised identities often continue to conceal (Major et al., 1990). The theme of concealment was also represented in the ‘masculinising’ symptoms of facial hair experienced by those with PCOS where such appearance-based concerns taboo, rather than symptoms that garnered sympathy like infertility. Affected individuals experienced strong societal expectations to engage in concealment practises, like hair removal, as a moral obligation whilst transgressions elicited shame (Lipton, Sherr, Elford, Rustin, & Clayton, 2006). Affected individuals were also found to embrace hypervigilant concealment practices regarding others seeing the perceived unacceptable aspects of their bodies (e.g. obesity in PCOS; vaginal scarring in VIN; breakthrough bleeding in fibroids; effects of pain in endometriosis) as a coping mechanism for managing these manifestations of their condition. These concealment practises were driven by attempts to hide their divergence from others, reflecting a stigmatised identity (McKinley & Hyde, 1996; Johnston-Robledo & Chrisler, 2013).

Given diagnostic delays of up to eight years are the norm amongst this BGC population (Seear, 2009), silence and concealment serve a protective function, sheltering individuals from the risk of invalidation that could incur when they make their private condition public. Moreover, withholding disclosure makes further sense when diagnostic delays for BGCs are often related to health professionals blaming symptoms on the affect individual’s mental health status (Culley et al., 2013; Gibson-Helm et al., 2016). Yet, as coping strategies most associated with poor health outcomes in chronic conditions are behavioural avoidance and low expression of emotions (Hagger & Orbell, 2003), in order to minimise the need for concealment it is important that clinicians respond to these body image-related concerns in a validating, rather than dismissive, manner and to refer affected individuals for appropriate psychological intervention when body image distress is apparent.

Within the reviewed studies the cultural differences theme highlighted the way in which normative expectations impact on willingness to disclose and the apparent need for concealment in order to avoid societal and familial ostracism. Infertility posed a particular issue in this instance, for those affected by BGCs living in non-Western cultures (Hadjiconstantinou et al., 2017; Markovic et al., 2008; Sharma & Mishra, 2018). Notably, one study which surveyed ethnically diverse participants with PCOS highlighted that the negative perception of symptoms (e.g. facial hair) varied across cultures (Hadjiconstantinou et al., 2017). These cultural differences in the interpretation of BGC symptoms and the experience of living with a BGC highlight the importance of actively including diverse ethnic groups in research on BGCs and body image, especially as prevalence rates of BGCs are higher in non-Caucasian ethnicities. Specifically, research indicates higher rates of PCOS in those from the Indian sub-continent, the Middle-East and Hispanic background (Fauser et al., 2012); two to threefold prevalence of uterine fibroids in African ethnicities compared to Anglo-Saxon women (Hellwege et al., 2017); and a ninefold rate of endometriosis in Asian ethnicities compared to other ethnicities (Sangi-Haghpeykar & Poindexter, 1995).

The feeling abnormal theme reflected fertility concerns, and for those with PCOS the ‘masculinising’ symptoms (e.g. facial hair) that challenged feminine identity of affected individuals, was discordant with other women in their lives and societal expectations. Unsurprisingly, many affected individuals expressed the desire for normality (i.e. absence of disease; Billhult & Stener-Victorin, 2012), and notably, this was less problematic for those who had children and were not planning to have more. However, the symbolism of the sex organs cannot be underrated, as female-identified individuals described feeling this was intricately linked to their identity, echoing cancer research whereby individuals with sex-organ disease experience considerable difficulties with body image changes (Baucom et al., 2008). Consideration of previous work on feminised physical bodies interacting and being constrained by gendered expectations within the culture is relevant to this theme (Piran, 2019).

Critically, the prevalence of accounts that reflected the theme of feeling abnormal signposts the incidence of high psychological distress in this population and dysfunctional coping strategies such as social withdrawal behaviours (e.g. not allowing a partner to see naked body due to embarrassment), as has been similarly found in some oncology populations (Fingeret, 2010). Across the reviewed studies and conditions many engaged in negative psychological self-appraisals and self-loathing, and experienced intrusive thoughts of bodily dissatisfaction. Taken together, these findings fit the ‘extreme’ pathological end of body image distress (Fingeret, 2010), and highlight a group of individuals not only managing the physical symptoms of BGCs but also being vulnerable for developing other psychological disorders including depression or anxiety. Given the extent of body image distress in these BGC populations, intensive psychological intervention is warranted, particularly since affected individuals are not typically actively seeking support for their body image issues.

The functional impact theme highlighted that body image is impacted by changes that occur to both physical functioning and appearance. The review found individuals had similar negative body image changes to that of cancer patients (Fingeret, Teo, & Epner, 2014). In this review, chronic pain, heavy bleeding and disability experienced by individuals affected with BGCs (e.g. endometriosis, fibroids, VIN) extended beyond appearance-limited concerns, also impacting the quality of life more generally, including sexual functioning and economic participation (Armour, Lawson, Wood, Smith, & Abbott, 2019; Jones et al., 2002). Reiterating the breadth of functionality is critical to understanding this population (Alleva & Tylka, 2021). Whilst PCOS had a wide-reaching symptom profile, the negative impacts on body image tended to be appearance based and were linked with impaired social participation. In particular, the functional impact of a BGC resulted in negative perceptions by others (e.g. romantic rejection), which may over time lead to long-term consequences of increased anxiety, reduced disclosure, and social withdrawal (Chaudoir & Fisher, 2010). Critically, across conditions, functional impacts reflected reduced quality of life, corroborating quantitative research indicating those living with BGCs often report lower health-related quality of life compared to unaffected populations (Jones, Hall, Ledger, & Balen, 2008).

## Clinical applications

Clinically, the widespread, chronic and largely hidden nature of body image concerns evident in BGC populations indicates a need for routine professional screening. Tools that have comprehensive conceptual coverage to multiple aspects of body image (e.g. social-consciousness, self-esteem, self-consciousness, ability to look at self, and noticeable change in appearance) are relevant, in the context of BGCs (Sundaram, Dhillon, Butow, Sundaresan, & Rutherford, 2019). Brief multidimensional standardised self-report measures such as The Derriford Appearance Scale (DAS24) (Carr, Moss, & Harris, 2005) and The Body Image Scale (Stead, Fountain, Napp, Garry, & Brown, 2004) may be suitable for regular use in clinical settings and can be administered by members of the healthcare team. These findings highlight the utility of clinical tools that assess for body image as imbued in functionality (Alleva & Tylka, 2021) as opposed to appearance and sensory aspects alone. Regular screening of body image concerns will allow for referral to appropriate mental health professionals in order to initiate early psychological intervention and support before these concerns risk impacting the identity of the affected individual. Moreover, research developing BGC-specific tools that measure the unique body image concerns of individuals with BGCs is crucial to facilitate detection and intervention for body image distress.

## Strengths, limitations and future research

The systematic scoping review is to our knowledge the first broad-scale analysis of qualitative research on body image experiences amongst individuals living with BGCs. A strength of the review was the inclusion of a comprehensive quality appraisal, which identified that most included studies were conducted to a high standard. Yet, these results should be understood in context of several limitations. In adopting a meta-synthesis approach of qualitative research, the interpretation of these findings was reliant on the researchers’ subjective interpretations (Lang et al., 2013), but this was mitigated by the stepwise, iterative and consultative process utilised by the reviewers (Noblit & Hare, 1988). Nevertheless, this qualitative is inherent subjectivity and the authors acknowledge the reanalysis of the same studies may produce different results. The quality appraisal revealed some weaknesses in the studies included in that most studies did not aim to explicitly examine experiences of body image concerns but more generally investigated aspects of relationship formation, psychological health, quality of life, and experiences of healthcare provision. Future research is needed that specifically targets the three broad domains of body image concerns in illness – appearance, sensory and functional (Fingeret, 2010). This review omitted grey literature in this emerging field, potentiating the exclusion of relevant unpublished work. Lastly, the criterion that only studies of individuals with diagnosed BGCs may potentially bias the range of included studies towards those investigating individuals with increased clinical severity, advanced course of disease, and with the acumen and resources to access medical help.

## Conclusion

In conclusion, this review demonstrates the centrality of body image-based concerns for those with BGCs. The chronicity and severity of individuals unique symptom profile is key to the type and intensity of body image concerns experienced. An overarching finding was that body image concerns are generally hidden, left untreated, and associated with reduced quality of life. Therefore, future research should consider investigating the barriers this population has in addressing their body image concerns. A greater understanding of how BGCs shape individual’s identity and relationship with their body will be of great benefit for those living with these common yet complex conditions.
